# Evaluation of fracture resistance among endodontically treated teeth using constant, variable and guiding taper endodontic file systems

**DOI:** 10.6026/973206300213420

**Published:** 2025-10-31

**Authors:** Sonam Bohra, Gurudutt Nayak, Maulsree Guleria, Drisya D Nambiar, Ejaz Ahmed, Manahil Qureshi, Simran Patil

**Affiliations:** 1 Department of Conservative Dentistry and Endodontics, Mansarovar Dental College, Bhopal, Madhya Pradesh, India

**Keywords:** Fracture resistance, vertical root fracture, ProTaper Ultimate, XP-endo rise, JIZAI, rotary file

## Abstract

Canal preparation is a critical step in endodontics because it involves removing a significant amount of dentin, which can weaken the root structure and lead to vertical root fractures. Therefore, it is of interest to evaluate the effect of rotary files with constant, variable and guiding tapers on the fracture resistance of endodontically treated teeth, as well as to assess the mode of fracture. Eighty freshly extracted human mandibular premolars were decoronated, simulated for periodontal ligament, embedded in acrylic resin blocks and subsequently instrumented using JIZAI, ProTaper Ultimate and XP-endo Rise systems. After obturation, fracture resistance was tested with a universal testing machine. The results showed that among the experimental groups, XP-endo Rise had the highest fracture resistance, which was statistically significant (p<0.05) compared to ProTaper Ultimate and JIZAI, indicating better preservation of dentin.

## Background:

The primary objective of endodontic treatment is to preserve natural teeth's form and function by removing potential infectious reservoirs through shaping, cleaning and sealing the root canal, ensuring a fluid-tight seal [1]. In the ever-evolving landscape of endodontics, the integrity and longevity of endodontically treated teeth (ETT) are major concerns. The strength of ETT is influenced by several predisposing factors, including excessive loss of tooth structure due to caries, trauma, access cavity preparation, dehydration of dentin with aging, the effect of various rotary files during instrumentation, the impact of different irrigating solutions and excessive pressure during filing procedures [1, 2]. Rotary systems with increased taper improve canal shaping and debridement but also remove more dentin and increase the risk of vertical root fracture (VRF) [1, 3]. When the force acting on the tooth exceeds the intrinsic strength of dentin, it leads to the formation of micro-cracking and craze lines, which eventually predispose to VRF [3]. It can occur before, during and after the root canal treatment [4]. It is the most unfavorable outcome in ETT, often leading to poor prognosis and inevitable extraction. But the taper is just a puzzle. Instrument design, cross-sectional shape and metallurgy also affect stress distribution on the tooth [5, 6]. To address this, Nickel-Titanium (NiTi) instruments have evolved with advances in cross-section, taper, metallurgy and kinematics [3, 5 and 6]. Concerning taper, there has been a paradigm shift, evolving from variable to constant and even to thermally responsive guiding tapers to improve efficiency and reduce procedural complications. The JIZAI is a newly developed NiTi rotary system by MANI, in Tochigi, Japan. This file system comprises a JIZAI orifice opener (25/0.14), a JIZAI Glider (13/.04) and shaping files of varying tip sizes, with a constant taper of .04 and .06%. The files are manufactured from a proprietary heat-treated NiTi alloy. Unlike conventional austenitic instruments, JIZAI contains R-phase and/or martensite phase at room or body temperature [7]. Dentsply Sirona's ProTaper Ultimate, a regressive taper file, builds on the legacy of the original ProTaper series, using a combination of three heat-treated NiTi alloys for M-Wire for the Slider (16/.02), Gold wire for the SX (20/.03), Shaper(20/.04), F1(20/.07), F2(25/.08) and F3(30/.09) files and Blue heat-treated wire for the FX (35/.12) and FXL (50/.10) files [8]. The XP-endo Rise file, designed by FKG Dentaire in Switzerland, is the latest addition to the XP-endo file series. It consists of two files, XP-endo Glider (15/.04) and XP-endo Rise Shaper (30/.04), both made of Max Wire technology [9]. Therefore, it is of interest to contribute to the growing body of literature on the effect of three distinct file systems with unique design features that may influence dentin preservation and fracture resistance of ETT, as well as the mode of fracture. The null hypothesis was tested that there is no significant difference in fracture resistance of ETT with these three file systems.

## Materials and Methods:

## Sample size calculation and standardization:

The sample size was calculated using G*Power software (version 3.1; Heinrich Heine University, Düsseldorf, Germany), with an effect size of 0.3, an alpha error of 0.05 and a statistical power of 80%. The calculation disclosed a total sample size for this study to be 80, with 20 samples in each group (n=20). 80 freshly extracted mandibular premolars for periodontal reasons, with a single straight root with a single patent canal and a completely formed apex, were selected. Teeth with dental caries, immature apices, resorption, any pre-existing cracks and previous endodontic treatment were excluded from the study. The samples were cleaned of adherent tissue tags and hard deposits using hand scalers and stored in 0.1% thymol until use. All the samples were decoronated using a diamond disc under water cooling to obtain a standardized root length of 16±1 mm from the anatomical apex. After sectioning, the working length (WL) was set by subtracting 1 mm short of the apex using a dental operating microscope (DOM) at 20x magnification with an ISO size #08 K-file visualized at the apical foramen. Buccolingual (BL) and mesiodistal (MD) dimensions were measured 7 mm from the apex, the cross-sectional area was calculated and the weight was measured in grams (gm). Samples were then evenly distributed into four groups (n=20) based on their cross-sectional area and weight (Table 1 - see PDF). Each root was wrapped in a single layer of aluminium foil and invested vertically in a plastic mold with self-curing acrylic resin, exposing 1mm coronally. Radiographs were taken to ensure the vertical positioning of each root. If the roots diverged, mounting was repeated. Subsequently, the aluminium foil was removed and the space filled with additional silicon impression material to simulate the periodontal ligament (PDL).

## Root canal instrumentation:

## Group 1: Control:

The root served as control and no root canal treatment was performed.

## Group 2: JIZAI:

All the roots were instrumented up to the WL using JIZAI rotary files to size 30/.06, following the sequence 25/.04, followed by 30/.04 and thereafter by 30/.06 files in a gentle pecking motion using crown-down single length technique at rotational speed of 500 rpm and torque 3 Ncm as per the manufacturer's instructions.

## Group 3: Protaper ultimate:

All the roots were instrumented using ProTaper Ultimate rotary files to size F3 beginning with Shaper (20/.04) file followed by the finishing files in sequence of F1 (20/.07) followed by F2 (25/.08) and thereafter by F3 (30/.09) files to the WL in a combination of brushing and pecking motions using crown-down single length technique at rotational speed of 400 rpm & torque 4 Ncm as per the manufacturer's instructions.

## Group 4: XP-endo rise:

All the roots were instrumented using an XP-endo Rise rotary file to size 30/.04. To begin, the file was cooled using Roeko Endo-Frost spray by Coltene-Whaledent, Langenau, Germany, inducing a phase transformation into martensite and reducing its taper to 30/.01. The instrument was then inserted into the canal, where exposure to body temperature (35°C) triggered a transition to the austenitic phase, causing the file to expand to its intended shape of size 30/.04. Then the file was advanced into the canal using five up-and-down long, gentle strokes to reach the WL at a rotational speed of 800 rpm & torque 1 Ncm as per the manufacturer's instructions. If the file failed to reach the WL from the first 5 strokes, the canals were irrigated with 5 mL of 2.5% sodium hypochlorite (NaOCl) and the procedure was repeated. Once the file reached the WL, 5 up-and-down movements were made with the file to complete the procedure. Each file was used to prepare four canals. During instrumentation, all files were lubricated with 17% ethylene diamine tetraacetic acid (EDTA) cream. Copious irrigation was done using 2 ml of 2.5% NaOCl using a 30-gauge side-vented needle in a syringe, between successive instrumentation. Finally, the canals were flushed with 5 mL of 17 % EDTA and 5 mL of 2.5% NaOCl for 1 minute, followed by 5 mL of saline. Subsequently, obturation was performed by single-cone and EndoSequence bioceramic sealer by Brasseler, USA, using the hydraulic condensation method. Then, the canals were sealed with a temporary filling material. All procedures were performed by a single operator to minimize variability and ensure consistency. Then the samples were stored at 37°C and 100% humidity for 7 days in an incubator for maturation.

## Fracture resistance testing:

All the prepared root samples were tested for fracture resistance with a universal testing machine (UTM). The compressive load was applied parallel to the long axis at the center of the root canal orifice of each root with a conical stainless-steel rod of 1mm in diameter and 60° taper tips at a crosshead speed of 1mm/min (Figure 1 - see PDF). The maximum force required to fracture each root was recorded in Newton (N) at the highest point of the load displacement curve from where the drop occurred. In most samples, this point was most often associated with an audible sound of a crack. After that, all the samples were examined under DOM at 20X magnification to record the mode of fracture, either in BL, MD, or as a compound fracture in both directions (Figure 2 - see PDF).

## Statistical analysis:

Statistical analysis was performed using SPSS version 26.0 (IBM, Chicago, IL, USA). The normality and homogeneity of variables, which included the BL and MD diameter, cross-sectional area and sample weights, were assessed using the Kolmogorov-Smirnov test. One-way ANOVA was used to compare the means of these variables and fracture resistance among groups, followed by Tukey's HSD post hoc test for intergroup comparisons. The chi-square test analyzed fracture modes. A p-value < 0.05 was deemed statistically significant.

## Results:

Based on the outcome of the fracture resistance test, the control group exhibited the highest fracture resistance (468.00 ± 18.81). While in the experimental groups, XP-endo Rise showed the highest fracture resistance (415.50 ± 28.00), followed by ProTaper Ultimate (372.00 ± 26.28) and the least fracture resistance was exhibited by JIZAI (342.50 ± 20.84) (Table 1 - see PDF). Among all the groups tested, the result exhibited a statistically significant difference (p<0.05). No significant differences in the mode of fracture were observed among the 4 groups (p>0.05). In overall distribution, BL fractures were the most prevalent across all groups, accounting for 70% of cases, followed by MD fractures at 16.25% and compound fractures were less frequently observed, at 13.75%.

## Discussion:

Endodontic treatment aims to manage pulpal and periapical diseases through shaping and cleaning [1]. However, instrumentation can cause stress and lead to dentinal microcracks, increasing the risk of VRF [3, 5]. VRF may originate at any root level and progress either coronally or apically, beginning at the buccal or lingual canal wall or extending to the external root surface can be complete or incomplete, often leading to BL fractures [10]. VRFs are multifactorial, influenced by tooth anatomy, teeth with narrow mesiodistal and wide buccolingual roots are more fracture-prone and by excessive dentin removal during canal preparation [3, 11 and 12]. Nearly 30% of ETTs exhibit fractures within five years, due to over-instrumentation [13] and approximately 10.9% are extracted due to VRF [14]. Apart from taper, tip size [12], cross-sectional design [5], type of alloy [6], kinematics of motion [13] and single or multi-file system [15], play a major role in the fracture resistance of the ETT. But the taper of the rotary file is crucial in determining the amount of dentin removed during shaping, potentially weakening the root [4, 12]. One can speculate that NiTi files with a smaller taper result in less dentinal structure loss and reduce the chance of VRF [16]. Hence, this study only focused on the impact of taper on the fracture resistance of the ETT. Moreover, it is indeed worth noting that the average biting force in the premolar region is 206 - 291 N, significantly lower than the fracture loads observed in most studies [4, 17]. This may clarify why VRF did not occur in every ETT. Therefore, this study aims to understand the effect of JIZAI, ProTaper Ultimate and XP-endo Rise with constant, variable and guiding taper on the fracture resistance of ETT. Rotary files with constant taper increase in diameter uniformly, while files with variable taper can increase, decrease, or alternate at different segments along the file length [18]. Guiding taper files have an adaptive core design made of Max-Wire alloy, allowing them to expand to three times their core diameter when subjected to specific body temperature [9]. This study focused on mandibular premolars as they are more prone to fracture post-endodontic treatment due to their thin dentinal walls and narrow MD dimensions [3]. Only straight-rooted premolars were included in this study to ensure standardized testing conditions, as root curvature can introduce stress concentrations during instrumentation and filling procedures [19]. Teeth were decoronated to a uniform root length to eliminate variations in instrumentation depth and obturation to ensure uniformity, reduce variability in force distribution and focus on only root structure, as supported by previous studies [16, 17 and 20]. Samples were stored in a 0.1% thymol solution due to its antimicrobial properties and minimal effect on dentin composition, while saline or purified water was avoided due to potential mineral loss of dentin and lack of antimicrobial properties [21]. A 2.5% NaOCl solution was used in shaping and cleaning procedures due to its potential effect on dentin. Higher concentrations of NaOCl solution significantly decrease the elastic modulus and flexural strength of dentin, whereas solutions of lower concentrations do not [22]. The study uses a single layer of aluminium foil with silicone-based elastomeric material to simulate the PDL, as the material dissipates occlusal forces and prevents microcracks [23], which is consistent with previous studies [20, 24]. In contrast, previous research has suggested that elastomeric materials cannot withstand compaction forces like natural ligaments, potentially leading to pressure collapse [25]. Hence, some studies omitted PDL simulation, while others used aluminium foil [2, 6] or modelling wax of varying thicknesses to create PDL space [18, 20 and 24]. This also reflects the difference observed in the fracture resistance among studies. A single-cone and bioceramic sealer passive obturation technique was employed because it minimizes mechanical stress on the canal walls and lowers the chance of fracture formation [26]. Conversely, the cold lateral compaction technique can introduce microcracks, especially in roots with thin dentinal walls and thereby increase the risk of VRF. Also, improper temperature control during warm vertical compaction of gutta-percha will cause the dentinal matrix to collapse [27].

The present study revealed that the uninstrumented control group had the highest fracture resistance (468.00 ± 18.81 N), consistent with previous findings [3, 4]. This finding validates that any intervention on dentinal walls tends to weaken the tooth structure. Variations in fracture resistance across studies may result from differences in tooth selection, dentin thickness, aging, PDL simulation and testing protocols, including sample size, storage conditions and mounting methods. All instrumented groups exhibited reduced fracture resistance, consistent with earlier reports [5, 12 and 13]. Among which, XP-endo Rise showed the highest resistance (415.50 ± 28.00 N), followed by ProTaper Ultimate (372.00 ± 26.28 N), while JIZAI recorded the lowest (343.50 ± 20.84 N). The post hoc comparison using Tukey's HSD test revealed statistically significant differences in fracture resistance across all groups (p<0.05); therefore, the null hypothesis was rejected, that following shaping with rotary files of constant, variable and guiding taper does not affect the fracture resistance of ETT. The XP-endo Rise, an advanced version of the XP-endo Shaper, has demonstrated the highest fracture resistance. This advanced system has a modified tip with six facets instead of three and an adaptive core design that begins shaping after a glide path of at least ISO 15, with a taper increasing from.01 to .04, reaching a final canal preparation of 30/.04. [9]. The superior performance of XP-endo Rise is not only attributed to its lesser taper but also due to its snake-shaped design and Max-Wire alloy metallurgy, which offers high flexibility and reduces the mechanical stress on canal walls, thereby decreasing the risk of crack initiation and propagation [28]. To substantiate these findings, Moriya *et al.* [29] analyzed stress distribution in simulated canals using the XP-endo Shaper and XP-endo Rise using a quasi-3D dynamic photoelastic method, finding no stress generation in the coronal third with the XP-endo Rise, while both files produced moderate stress in the middle and apical thirds. ProTaper Ultimate files offer moderate fracture resistance compared to XP-endo Rise, while JIZAI files exhibited the lowest. They have rhomboidal cross-section beginning from D1 to D3 and evolve to an ever-changing partially off-centred parallelogram form with acute angles at different lengths of the instruments from D4 to D16. This enables precise adjustment of each file segment's cutting efficiency based on the expected workload in certain areas. The heat-treated gold wire and proprietary alternating offset machining process enhance the file's flexibility and reduce the contact between the file and dentin, thereby minimizing the cutting stress on dentin [30]. The Shaper of this system is a single "super-shaper" file that combines the functions of S1 and S2 and it is manufactured from 1.0 mm NiTi wire, compared to the 1.2 mm wire of traditional ProTaper files. The file has a minimally progressive taper over its active portion with cross-sectional diameters at D0, D4, D8, D12 and D16 of 0.20 mm, 0.40 mm, 0.65 mm, 0.88 mm and 1.0 mm, respectively, which further contributes to conservative dentin removal. The finishers are specifically designed with a progressive taper from D1 to D3, which primarily focuses on the concept of deep shape for apical cleaning and then taper regresses from D4 to D16. The Shaper dominantly prepares the coronal two-thirds of a canal, whereas the Finishers enlarge the apical third without over-preparing the canal body, preserving coronal dentin. This design balances both shaping efficiency and structural conservation, but moderate dentin removal may reduce fracture resistance compared to XP-endo Rise.

In contrast, the JIZAI system demonstrated the lowest fracture resistance despite its sophisticated design of off-center quasi-rectangular cross-section and sharp cutting edges [7]. The aggressive cutting characteristics, although beneficial for shaping efficiency, appear to remove more dentin and induce stress on the canal walls. Besides this, the last file used in the study was 30/.06 with a constant taper designed over its active portion with cross-sectional diameters at D0, D4, D8, D12 and D16 of 0.30 mm, 0.54 mm, 0.78 mm, 1.02 mm and 1.26 mm, respectively. In comparison to the ProTaper Ultimate Shaper, this file has a wider cross-sectional area, which is predominantly responsible for uniformly widening the canal and reducing the thickness of coronal dentin and may result in over-instrumentation, especially in the cervical third and increasing vulnerability to VRF. All the NiTi files in this study were used in a continuous rotational motion to standardize the sample preparation. There is a lack of direct comparisons between JIZAI, ProTaper Ultimate and XP-endo Rise File on the fracture resistance of ETT. Microcrack formation is associated with VRF [6] and Çiçek *et al.* [20], also reported that rotary instruments induce more microcracks due to higher dentinal strain. However, no significant difference in fracture resistance exists between teeth treated with rotary or reciprocation motion [6, 16]. Kim *et al.* [5] in their finite element analysis stated that greater tapered files because increased stress on the canal walls, thus decreasing the fracture resistance of ETT. However, some studies have reported contradictory findings, suggesting that the taper of the file does not significantly affect fracture resistance [18, 31]. The study found that most ETT samples fractured in the BL direction, followed by the MD direction, with compound fractures less common. This aligns with previous findings, as stresses were oriented more in the BL direction [20] regardless of the thickness of the proximal dentin wall [11]. This study provides valuable insights but has limitations due to its *in vitro* conditions, like a lack of bone support, biological responses, dynamic physiological stresses and motion of instrumentation. It also neglects evaluation of factors like microcrack formation and residual dentin thickness. Despite these limitations, the study offers useful comparative data but has limited clinical relevance and requires long-term *in vivo* studies for validation.

## Conclusion:

This study provides novel insights, demonstrating that although all rotary instrumentation systems reduce the fracture resistance of ETT. XP-endo Rise preserves tooth strength significantly better than JIZAI and ProTaper Ultimate. Thus, we show the importance of choosing file systems that not only shape canals effectively but also preserve critical dentin.
